# Supplementary material for: Multifocal Neuroblastoma and Central Hypoventilation in An Infant with Germline ALK F1174I Mutation
Source: Diagnostics (Basel). 2022 Sep 19;12(9):2260. doi: 10.3390/diagnostics12092260 (PMC9498070; doi:10.3390/diagnostics12092260)
Supplement: Supplementary file 1 [file diagnostics-12-02260-s001.zip › diagnostics-1910338-supplementary.pdf]

Table S1. Comparison of present case with cases as reported in de Pontual et al [31].

	Case Presented	Case 1 - de Pontual et al	Case 2 - de Pontual et al
**Sex**	female	female	female
**Gestastional age**	32 weeks	full term	31 weeks
**Birth Weight**	1890 g (-0,5 SD)	3100 g	1300 g
**Birth Length**	43 cm (+/-0 SD)	46 cm	not specified
**Head Circumference**	31.5 cm (+1SD)	34 cm	28.5 cm
**Growth**	poor overall growth, deceleration of head growth	deceleration of head growth	poor overall growth, deceleration of head growth
**Age at NB diagnosis**	4 weeks	3 days	3 weeks
**NB presentation**	multifocal lobular masses in abdomen and thorax	adrenal NB with pelvic extension, rapid tumor progression	bilateral large heterogenous and calcified adrenal masses
**NB histology**	undifferentiated in thoracic tumor, ganglioneuroblastoma in abdomen	not specified	infiltrating islands of undifferentiated neuroblasts.
**NB genetic profile**	non-MNA, numerical only (sample 1&2), other segmental aberrations (sample 3)	non-MNA, non-1p-del (FISH)	non-MNA, 4 copies of MYCN, trisomy 1, trisomy 9, tetrasomy 17 (FISH)
**Germline *PHOX2B* mutation**	no	no	no
**Germline *ALK* mutation**	F1174I, *de novo*	F1245V, *de novo*	F1174V, *de novo*
**Congenital Malformations**	diaphragmatic hernia, unilateral inguinal hernia	(high arched palate)	tracheobronchomalacia, bilateral inguinal hernias, patent foramen ovale
**Intestinal Findings**	hypomotile; no abnormal enteric plexuses	hypomotile; no abnormal enteric plexuses	hypomotile; no abnormal enteric plexuses
**Autonomic Instability**	frequent episodes of apneas, hypoventilation and desaturation	episodes of apneas and desaturation	daily episodes of desaturation; temperature instability
**CCHS workup**	polysomnography: apneas independent of epileptic activity; reduced heart rate variability consistent with CCHS	CCHS initially suspected but ruled out due to independence from sleep-awake state and absence of *PHOX2B* mutation	CCHS initially suspected but ruled out due to independence from sleep-awake state and absence of *PHOX2B* mutation
**Neurologic Symptoms**	atrophic muscles, abnormal movements, contractures, possibly deviant eyesight behaviour; neurogenic bladder dysfunction; intact sucking reflex	hypotonia, scarce spontaneous movement, absence of sucking and swallowing reflexes	hypotonia, scarce spontaneous movement, poor sucking, swallowing difficulties; abnormal movements of right arm and leg
**EEG findings**	epileptiform activity but clinical seizures without corresponding EEG changes	slow activity; no signs of epilepsy	no epileptiform activity
**Brain MRI**	normal brain MRI including brainstem	abnormal shape of brainstem	abnormal shape of brainstem
**Cause of Death**	respiratory failure	apnea; no attempt at resuscitation	withdrawal of intensive care
**Age at Death**	3 months	4.5 months	9 months
